# Functional and biophysical characterization of an HLA-A*6801-restricted HIV-specific T cell receptor

**DOI:** 10.1002/eji.200636243

**Published:** 2007-02

**Authors:** Emma Gostick, David K Cole, Sarah L Hutchinson, Linda Wooldridge, Sabrina Tafuro, Bruno Laugel, Anna Lissina, Annette Oxenius, Jonathan M Boulter, David A Price, Andrew K Sewell

**Affiliations:** 1 Nuffield Department of Medicine, University of Oxford, John Radcliffe HospitalOxford, UK; 2 Department of Medical Biochemistry and Immunology, Cardiff University School of MedicineCardiff, UK; 3 Institute for Microbiology, ETH HönggerbergZürich, Switzerland

**Keywords:** HIV-1, HLA-A68, TCR

## Abstract

HLA-A*6801 exhibits several unusual features. First, it is known to bind weakly to CD8 due to the presence of an A245V substitution in the α3 domain. Second, it is able to accommodate unusually long peptides as a result of peptide ‘kinking’ in the binding groove. Third, CD8^+^ cytotoxic T lymphocytes that recognise HLA-A*6801-restricted antigens can tolerate substantial changes in the peptide sequence without apparent loss of recognition. In addition, it has been suggested that HLA-A68-restricted TCR might bind with higher affinity than other TCR due to their selection in the presence of a decreased contribution from CD8. Here we (1) examine monoclonal T cell recognition of an HLA-A*6801-restricted HIV-1 Tat-derived 11-amino acid peptide (ITKGLGISYGR) and natural variant sequences thereof; (2) measure the affinity and kinetics of a TCR/pHLA-A68 interaction biophysically for the first time, showing that equilibrium binding occurs within the range previously determined for non-HLA-A68-restricted TCR (KD approx. 7 μM); and (3) show that “normalization” of the non-canonical HLA-A*6801 CD8-binding domain enhances recognition of agonist peptides without inducing non-specific activation. This latter effect may provide a fundamental new mechanism with which to enhance T cell immunity to specific antigens.

## Introduction

The MHC class I (MHCI) antigen processing pathway presents short peptides, derived from endogenously expressed proteins, at the cell surface 1. The highly variable complementarity-determining regions of TCR on the surface of CD8^+^ cytotoxic T lymphocytes (CTL) interact with both the peptide and the peptide-binding platform of the MHCI molecule to confer peptide-MHCI (pMHCI) specificity 2. Almost all nucleated cells express MHCI, thereby enabling CTL to scan for internal anomalies. The ability of CTL to detect and destroy cells displaying aberrant-self or non-self peptides makes them key determinants of immunity to neoplastic cells and intracellular pathogens.

Other ‘accessory’ molecules at the T cell surface also contribute to antigen recognition in concert with the TCR. Some of these molecules act to produce co-stimulatory signals while others serve to aid cellular adhesion. Notably, the recognition of T cell antigens by the αβ TCR is unique among receptor-ligand interactions as it involves engagement of a ‘co-receptor’ molecule that binds to the MHC at a largely invariant site distal from the TCR-docking platform 3–5. The co-receptor for MHCI antigens, CD8, is known to facilitate the process of antigen recognition by a number of mechanisms 6–8.

Human CD8 makes contacts with the MHCI α3 domain, particularly the α3 ‘loop’ (residues 225–232), the underside of the α2 domain (residues 115, 122, 128 and 198) and β2-microglobulin 3. This region is largely invariant and human MHCI molecules are known to exhibit similar affinities for CD8 (KD=100–200 μM) 9. A very small minority of human MHCI molecules, HLA-A*68, -B*48 and -B*81, differ in their α3 domain sequence. In HLA-A*68, a valine residue at position 245 replaces the standard alanine present in other MHCI molecules; in HLA-B*48 and -B*81, residue 245 is a threonine. Molecular modeling based on the structures of HLA-A*0201, HLA-A*6801 and the HLA-A*0201/CD8 cocrystal predicts that the larger valine residue at position 245 in HLA-A*6801 distorts the α3 loop of the molecule, resulting in a less energetically favorable interaction with CD8 3, 9. This is presumably also true for the threonine residue at position 245 as HLA-B*4801 shows greatly reduced CD8 binding (KD>1000 μM) 9. Substitution of the canonical alanine residue at position 245 of HLA-A*0201 for valine or threonine confirms the effects of these mutations and increases the KD of CD8 binding from approximately 130 μM to 500 μM and 470 μM, respectively 9.

HLA-B*48 and -B*81 are uncommon in Caucasian populations and CTL responses restricted by HLA-B*48 and -B*81 molecules have yet to be studied in detail. HLA-A*68-restricted CTL, especially those restricted by HLA-A*6801, have been of interest for many years due to the reduced CD8 binding of this molecule. Indeed, studies of HLA-A*6801 (formerly known as HLA-Aw68.1) have greatly improved our understanding of pMHCI 10–12.

HLA-A*6801 (referred to as HLA-A68 from hereon) is known to bind CD8 very weakly (KD∼1000 μM) 9, 13. In addition to reduced CD8 binding, HLA-A68 exhibits other unusual features. First, it is able to restrict unusually long peptide antigens as a result of ‘kinking’ in the binding groove 11. Second, HLA-A68-restricted CTL are capable of accepting a remarkable degree of residue substitution in the bound peptide 14. A CTL line specific for the influenza epitope KTGGAIYKR was able to recognise efficiently a peptide alanine-substituted at all but a single non-MHC anchor position (ATAAAIAAR) 14. Just 25 million human TCR 15 have to cover all the possible MHC-restricted peptides generated from permutations of the 20 proteogenic amino acids. As a result, an extremely high level of cross-reactivity is essential for effective T cell immunity 16. Our recent findings show that the MHCI/CD8 interaction controls optimal levels of T cell cross-reactivity (Laugel *et al*., in preparation). Indeed, introduction of the A245V substitution in HLA-A*0201 substantially reduces the number of variant antigens that can be recognised by HLA-A*0201-restricted CTL clones (Laugel *et al*., in preparation) and we suggest that HLA-A68-restricted TCR might incorporate increased binding degeneracy in order to compensate for their weakened CD8 binding and allow coverage all potential HLA-A68-restricted peptides. Third, it has been suggested that HLA-A68-restricted TCR might bind cognate antigen with higher affinity than those restricted by MHCI molecules with a canonical CD8-binding domain due to their thymic selection in the presence of a reduced pMHCI/CD8 interaction 17.

Herein, we measure the affinity and kinetics of a TCR/pHLA-A68 interaction biophysically for the first time. In addition, we normalize the non-canonical CD8-binding domain of HLA-A68 and undertake the first study of the biological effects of increasing the affinity of the pMHCI/CD8 interaction while TCR/pMHCI interactions remain faithful.

## Results

### Recognition of natural HIV-1 variants by HLA-A68-resticted Tat-specific CTL

SC21, a patient with primary HIV-1 infection, made a dominant CTL response to the patient-autologous Tat-derived peptide ITKGLGISYGR through HLA-A68. Residue 2 and the C-terminal residue act as anchors for HLA-A68 11. Neither of the two 10-mer peptides contained within the 11-mer ITKGLGISYGR epitope were antigenic 18. Consequently, this HIV-1 Tat-derived peptide is longer than normal MHCI-presented peptides and is expected to bulge out from the HLA-A68 α1/α2 peptide-binding platform. Such bulging of peptides bound to HLA-A68 has previously been documented 11. The patient-autologous Tat epitope sequence (ITKGLGISYGR) differed from the HIV-LAI reference sequence at positions 1 and 4 (**T**TK**A**LGISYGR). A CTL line grown from a SC21 sample taken at day 297 following the onset of symptoms was used to produce a vigorously growing CTL clone (c23).

**Figure 1:**
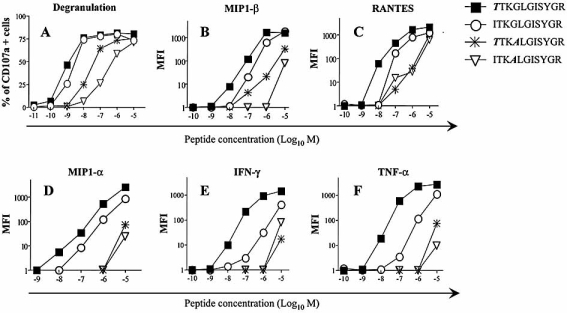
Activation profile of CTL clone c23 in response to various agonist ligands. Six different read-outs were used to assess the response of c23 CTL to four different agonist peptides in a 4-h dose-response assay; cells and supernatants were harvested separately. (A) The level of degranulation marker CD107a up-regulation on CD8^+^ cells was determined by flow cytometry. (B–F) Release of 11 cytokines and chemokines in the cell supernatant was estimated with bead arrays; substantial quantities were detected for 5/11 of these lymphokines.

At the first time point that patient virus was sequenced (day 60 following the onset of symptoms), four out of 13 clones encoded a T at position 1 of the ITKGLGISYGR epitope 19. The 1T sequence was absent at the next sequencing time point (day 327 following the onset of symptoms). Thus, it is possible that the founder virus in SC21 encoded the 1T sequence. We analyzed responses to the patient ‘index’ sequence (ITKGLGISYGR), the LAI reference sequence (**T**TK**A**LGISYGR), the patient variant (**T**TKGLGISYGR) and the intermediate (ITK**A**LGISYGR) sequence (Fig. 1). For the sake of ease, these peptides are described with reference to the position within the patient epitope that is modified (*i.e.* as ‘index’, 1T/4A, 1T and 4A, respectively) from hereon.

The 1T variant was consistently recognised at lower concentrations than the ‘index’ peptide in functional assays of degranulation and cytokine/chemokine release (Fig. 1). The difference in activation potential between the index and 1T sequences varied between effector functions (Fig. 1), being minimal for degranulation, intermediate for MIP-1α/β and greatest for IFN-γ and TNF-α production; this pattern conforms to the level of TCR triggering required to induce these effector functions (degranulation/killing < MIP < IFN-γ) 20, 21. The fact that the 1T variant acts as the best agonist may also indicate that this sequence represents the founder virus that initially induced the HLA-A68-restricted Tat response in SC21, although retrospective confirmation of this is not possible.

### Manufacture and biophysical analysis of soluble HLA-A68-restricted TCR

**Figure 2:**
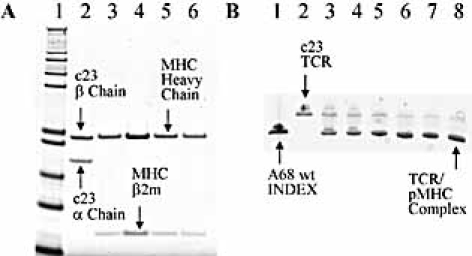
The quality and biological activity of the proteins used in this study. (A) SDS-PAGE purity analysis of c23 TCR and each A68 variant. The numbered lanes on the gel are as follows: 1: Seeblue molecular markers; 2: c23 TCR; 3: A68 WT index; 4: A68 WT 1T; 5: A68 245A index; 6: A68 227/8KA index. (B) Native gel analysis of complex formation between c23 TCR and A68 WT index. Different molar ratios of c23 and A68 were mixed together in order to determine the optimum conditions for complex formation. The numbered lanes on the gel are as follows: 1: A68 WT index; 2: c23 TCR; 3: c23 TCR 4:1 A68 WT index; 4: c23 TCR 3:1 A68 WT index; 5: c23 TCR 2:1 A68 WT index; 6: c23 TCR 1:1 A68 WT index; 7: c23 TCR 1:2 A68 WT index; 8: c23 TCR 1:3 A68 WT index. As the ratio of A68 WT index increases compared to c23 TCR, the amount of c23 TCR forming the complex also increases. At a molecular ratio of c23 TCR 1:1 A68 WT index, >90% of c23 TCR has formed the complex.

**Figure 3:**
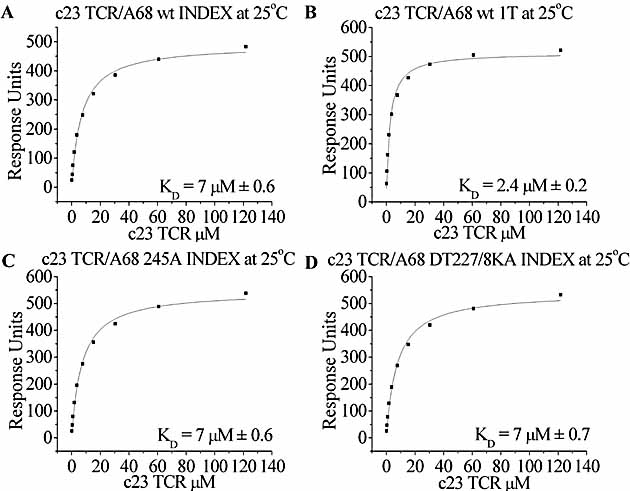
Surface plasmon resonance of HLA-A68-restricted c23 TCR. Equilibrium binding responses at 25ºC of the c23 TCR against: (A) A68 WT complexed to index (ITKGLGISYGR) peptide, (B) A68 WT complexed to 1T (TTKGLGISYGR) peptide, (C) A68 245A complexed to index peptide and (D) A68 227/8KA complexed to index peptide. Ten serial dilutions were carried out in triplicate for each equilibrium experiment. The average response for each concentration is plotted with standard deviation (*n*=3). The equilibrium binding constant (KD) values are plotted using a nonlinear curve fit [*y* = (P_1_*x*)/(P_2_ + *x*)[. c23 TCR binding to each A68 variant with the index peptide was identical with a KD of 7 μM. c23 TCR bound about three times stronger to the A68 WT 1T complex with a KD of 2.4 μM. Introducing the DT227/8KA or the V245A mutations into HLA-A68 abrogates (KD>10 000 μM) or enhances (KD=102 μM) the already weak HLA-A68/CD8 interaction (KD = 980 μM), respectively 13, without affecting the affinity of the TCR/pMHCI interaction. These mutations therefore do not disrupt the α1/2 peptide-binding platform of the molecule that is recognised by the TCR.

**Figure 4:**
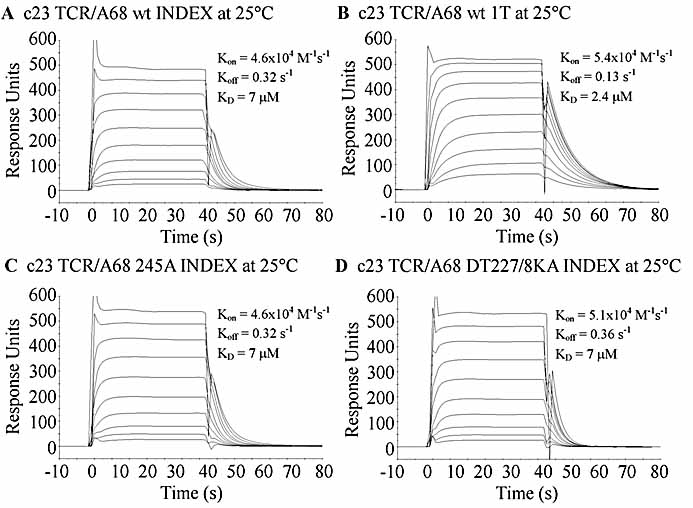
Kinetic binding analysis at 25ºC of the c23 TCR. Binding to: (A) A68 WT complexed to index peptide, (B) A68 WT complexed to 1T peptide, (C) A68 245A complexed to index peptide and (D) A68 227/8KA complexed to index peptide. Ten serial dilutions of concentrated TCR were injected at 30 μL/min for 45-s association periods. The solid lines for each binding response were calculated assuming 1:1 Langmuir binding [AB = B*AB_MAX_/(KD + B)] and the data were analyzed using a global fit algorithm (BIAevaluation^TM^ 3.1) to calculate K_on_ and K_off_ values. The c23 TCR kinetics for each A68 variant with the index peptide were virtually identical with K_off_ values of 0.32–0.36 s^–1^ and K_on_ values of 4.6×10^4^ M^–1^s^–1^ and 5.1×10^4^ M^–1^s^–1^. For the A68 WT 1T complex, the c23 TCR bound with a K_on_ similar to that of the index-complexed A68 variants of 5.4×10^4^ M^–1^s^–1^. The stronger affinity observed was due to an extended K_off_ of 0.13 s^–1^.

The TCR from c23 was manufactured as a soluble protein; the sequence of the TCR alpha and beta chains highlighting the complementarity-determining region loops is shown in Supplementary Fig. 1. All the proteins utilized in this study were of high purity (Fig. 2A). Gel-shift analysis with soluble c23 TCR and HLA-A68-ITKGLGISYGR showed that >90% of the c23 TCR bound to its cognate ligand (Fig. 2B). Surface plasmon resonance analysis showed that the two antigens that occurred in the patient, HLA-A68-ITKGLGISYGR and HLA-A68-TTKGLGISYGR, bound to c23 TCR with KDs of 7.0±0.68 μM and 2.5±0.2 μM, respectively (Fig. 3). These affinities are within the normal range (KD=1–10 μM) 2 that we have observed for eight antiviral HLA class I-restricted TCR (Cole *et al*., in preparation). That the c23 TCR bound to the 1T peptide with higher affinity than the index peptide, attributable primarily to a slower off-rate (Fig. 4), is consistent with the fact that this sequence is a better agonist for c23 CTL according to current kinetic models of T cell activation (Fig. 1) 22, 23.

### Enhancement of the HLA-A68/CD8 interaction

We manufactured two mutated forms of HLA-A68 in order to increase or abrogate CD8 binding. We have previously examined CD8 binding to HLA-A68 13, 18. WT HLA-A68 binds CD8 with low affinity (KD=980 μM) 13. A double substitution of D_227_ and T_228_ to K and A, respectively (DT227/8KA), abrogates the interaction with CD8 (KD>10 mM), while a V_245_-to-A (V245A) substitution increases the affinity of this interaction (KD=102 μM) 13. Multimeric forms of DT227/8KA HLA-A68, WT HLA-A68 and CD8-enhanced (V245A) HLA-A68 antigens all bind to cell surface TCR equally well, suggesting that these substitutions do not interfere with the α1/α2 peptide-binding platform of the HLA-A68 molecule contacted by the TCR (13 and data not shown). In order to confirm that these mutations do not affect TCR binding, we performed surface plasmon resonance experiments using HLA-A68, DT227/8KA HLA-A68 and V245A HLA-A68 folded with the ITKGLGISYGR index peptide. All three HLA-A68 antigens bound the c23 TCR with equivalent affinity (KD=7 μM; Fig. 3A, C, D) but showed the expected differences in the binding of CD8αα molecules (13 and data not shown).

### Biological effects of an enhanced HLA-A68/CD8 interaction

**Figure 5:**
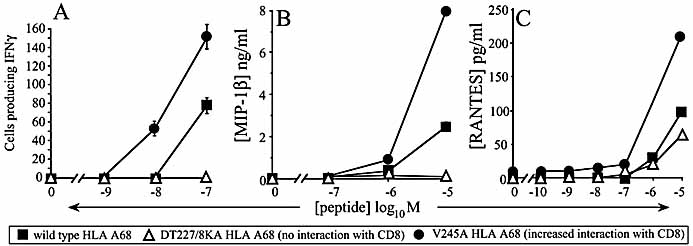
Increasing the HLA-A68/CD8 interaction enhances T cell sensitivity and response magnitude. C1R target cells expressing identical levels of WT HLA-A68, DT227/8KA HLA-A68 or V245A HLA-A68 were pulsed with the indicated concentrations of peptide and used to stimulate c23 ITKGLGISYGR-specific CTL. Activation read-outs were IFN-γ ELISPOT (A) using 2.5×10^2^ CTL and 2.5×10^4^ C1R transfectants at 37°C in a 4-h assay, and MIP-1β (B) or RANTES (C) ELISA 20 of supernatant after 2.5×10^4^ CTL were incubated with 2.5×10^4^ C1R transfectants at 37°C for 4 h. Assays were performed as described previously 13. Error bars show standard deviation from the mean of three replicate assays, although in most cases the error is smaller than the plot symbol.

**Figure 6:**
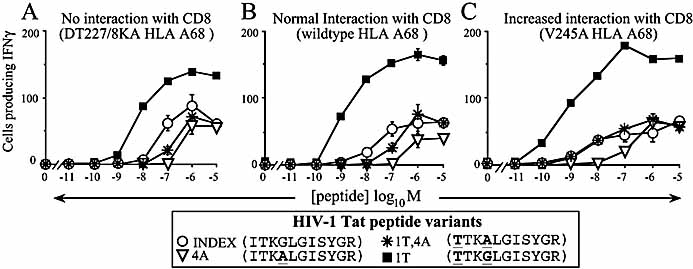
Increasing the HLA-A68/CD8 interaction does not alter the CTL recognition pattern of peptide variants. HLA-A68-restricted c23 ITKGLGISYGR-specific CTL (10^3^) were incubated with 2.5×10^4^ C1R transfectants in a 3.5-h ELISPOT assay. Targets expressing identical levels of DT227/8KA HLA-A68 (A), WT HLA-A68 (B) or V245A HLA-A68 (C) were pulsed with indicated amounts of index peptide or naturally occurring variants as shown in the key. Error bars show standard deviation from the mean of three replicate assays, although in most cases the error is smaller than the plot symbol.

The ability to ‘normalize’ CD8 binding of HLA-A68 by V245A substitution provided a unique opportunity to examine the biological effects of enhanced CD8 binding for the first time. Full-length DT227/8KA HLA-A68, WT HLA-A68 and V245A HLA-A68 were expressed in C1R cells by stable transfection using previously described methodology 13. Peptide-pulsed targets expressing HLA-A68 with enhanced CD8 binding were recognised by cognate CTL at lower antigen concentrations and induced greater production of IFN-γ, MIP-1β and RANTES from these CTL at equivalent peptide concentrations compared to target cells expressing WT HLA-A68 (Fig. 5). Enhancing CD8 binding did not alter the recognition pattern of natural HIV-1 Tat variants (Fig. 6). Thus, increasing the interaction between HLA-A68 and CD8 results in a marked improvement in the functional recognition of cognate antigen. Importantly, our data (Fig. 5, 6 and not shown) show no background activation of CTL by targets expressing V245A ‘CD8-enhanced’ HLA-A68 and rule out the possibility that c23 is able to recognise natural levels of self peptides presented in the context of this molecule.

## Discussion

In this report, we describe the manufacture and characterization of the first HLA-A68-restricted TCR. This TCR was shown to bind to cognate antigen with an affinity well within the normal range for anti-viral TCR restricted by other MHCI molecules. Thus, the selection of TCR in the presence of impaired CD8 binding need not result in supranormal TCR/pMHCI affinities. At 11 amino acids, the HLA-A68-restricted peptide recognised by the c23 TCR is unusually long for MHCI-restricted antigens and is believed to kink away from the peptide binding platform 11. This may necessitate unusual TCR docking as has recently been described for a super-bulged 13-amino acid Epstein–Barr virus-derived peptide restricted by HLA-B*3508 24. The requirement for the unusual docking associated with bulging peptides may bias the TCR repertoire and result in limited TCR diversity 25. The realization that peptides of greater than ten amino acids in length can be presented by MHCI molecules and recognised by cognate TCR has led to recent calls to broaden the algorithms widely used to predict MHCI-binding peptides 26. To date, there has been no systematic and unbiased comparison between HLA-A68, -B35 and other MHCI molecules and it is therefore not possible to know whether HLA-A68 and -B35 are unusual in binding long peptides.

We found that “normalizing” the CD8 binding of HLA-A68 resulted in enhanced recognition of agonist peptides without resulting in recognition of self peptide-HLA-A68 complexes (Fig. 5, 6). CD8-enhanced pMHCI ligands were recognised at lower concentration and induced substantially better effector function for a given quantity of antigen (Fig. 5, 6). CD8 is known to facilitate the process of antigen recognition by a number of mechanisms 6–8. To date, it has been established that CD8 (1) recruits the TCR to specific membrane domains believed to be privileged sites for TCR-mediated signal transduction 27; (2) recruits essential signaling molecules to the intracellular side of the TCR/CD3/ζ complex 28, 29; and (3) stabilizes the TCR/pMHCI interaction at the cell surface 30, 31. It is not yet clear whether CD8 has other roles in antigen recognition. CD8-mediated topographical TCR organisation is thought to be a function of a direct interaction between these molecules 27, 32, 33 and is not therefore likely to depend on the pMHCI/CD8 interaction. Thus it is likely that the effects we observe are the result of enhanced TCR dwell times and superior intracellular signaling.

Our recent experiments with HLA-A2 predict that V245A substitution of HLA-A68 will enhance TCR mean dwell time by ∼15% more than that afforded by a WT interaction 31. It is therefore likely that there will be some ligands that fall within the narrow window of TCR/pMHCI dwell times that allow them to be ‘null’ in the context of HLA-A68 but weak agonists in the context of V245A HLA-A68. Such ligands are likely to be rare amongst the myriad of possible HLA-A68-restricted peptides encoded by the 20 proteogenic amino acids. That V245A HLA-A68-expressing targets do not activate c23 CTL in the absence of added peptide is good evidence that there are no self-expressed ligands for this TCR that occur within this narrow affinity window.

In summary, our results indicate that there may be scope for improving the recognition of HLA-A68-restricted antigens. We have recently demonstrated that ‘CD8 enhancement’ can be engineered into any human MHCI molecule and, therefore, any MHCI-restricted antigen 31. Experiments are underway to determine whether the improved antigenicity of ‘CD8-enhanced’ antigens is a general phenomenon. Our findings suggest a novel mechanism that could enable boosting of specific cytotoxic immunity, an approach that might be especially pertinent in the context of anti-tumor CTL responses. In addition, CD8 enhancement might prove useful in the setting of vaccination strategies to enhance the generation of pMHCI-restricted peptide-specific CTL responses.

## Materials and methods

### Cloning of HLA-A68-restricted HIV-1 Tat-specific CTL

Patient SC21, a 37-year-old homosexual male, presented with primary HIV-1 infection shortly after a well-documented high-risk sexual exposure 19. The patient mounted substantial T cell responses to four MHCI-restricted antigens as quantified by IFN-γ ELISPOT in the 10-month period following the onset of symptoms 19. The numerically dominant response (5000–8000 spot-forming cells/10^6^ peripheral blood lymphocytes) was specific for an epitope from the HIV-1 Tat transactivator protein 19. SC21 peripheral blood lymphocytes were used to map this epitope directly *ex vivo* with an adapted IFN-γ ELISPOT assay and a library of eight 20-amino acid peptides from the Tat protein that overlapped by ten residues 18. An HLA-A68-restricted response to the Tat4 peptide (Tat residues 31–50, FHCQVCF**T**TK**A**LGISYGRKK) was observed.

Subsequent sequence analysis of the patient's autologous virus identified two amino acid changes compared to the HIV-LAI reference sequence that was used to make the peptide library (38I and 41G) 19. Responses to the autologous Tat4 peptide were consistently better than to the HIV-LAI sequence 18. Further analysis, using the patient-autologous sequence, narrowed down the peptide antigen to an 11-amino acid epitope (ITKGLGISYGR) 18. Residue 2 and the C-terminal residue act as anchors for HLA-A68 11. Neither of the two 10-mer peptides contained within the 11-mer ITKGLGISYGR epitope were antigenic 18. A CTL line was grown from a SC21 sample taken at day 297 following the onset of symptoms. This CTL line was then cloned by limiting dilution at 0.3 cells/well to produce a vigorously growing CTL clone (c23).

### Target cells

The manufacture of C1R cells expressing HLA-A68 is described elsewhere 13. Cells were cloned and tested with relevant antibodies to ensure that they expressed identical levels of MHCI on their surface.

### Surface plasmon resonance, soluble pMHCI and TCR manufacture

Soluble pMHCI manufacture, tetramerization and biophysical studies were performed as previously described 31. The mutations in HLA-A68 and the biophysical validation of their effects are published elsewhere 13, 31. In order to obtain the sequence of an HLA-A68-restricted TCR, cDNA from c23 was used as a template in 42 separate PCR using a primer set to cover all TCRAV and TCRAJ genes. Only one reaction generated a product. Sequencing confirmed a TCR α chain made from the TCRAV 14 gene with a TCRAJ 20 joining region (IMGT nomenclature). The TCRB sequence was generated by a single PCR with a combined primer set 34. The reaction yielded a single product. Sequence analysis showed a TCRBV 7-9 gene with a TCRBJ 1-1 joining region. The TCR α chain was cloned into pGMT7 expression vector as a fusion construct with the c-jun leucine zipper region 35. A TCR β chain pGMT7 expression vector was constructed to express the TCR chain as a fusion with the v-fos leucine zipper 35. Expression vectors were transformed into *Escherichia coli* Rosetta DE3 (pLysS) and protein was produced as inclusion bodies by inducing protein expression with 0.5 mM isopropyl-β-d-thiogalactopyranoside.

Following cell harvest by centrifugation, inclusion bodies were isolated by sonication and purified with three successive detergent washes using 0.5% Triton X-100. Inclusion bodies were given a final wash in resuspension buffer to remove any detergent before being resolubilized in guanidine solution (6 M guanidine, 50 mM Tris pH 8.1, 100 mM NaCl, 10 mM EDTA, 10 mM dithiothreitol). Insoluble material was pelleted by centrifugation and the supernatant stored at –80°C. TCR-zipper chains were refolded at a 5:1 ratio of α:β chain. Each solubilized inclusion body chain was diluted to 5 mg/mL in guanidine solution. To ensure complete denaturation, dithiothreitol was added to a concentration of 10 mM and chains were incubated at 37°C for 30 min. Refolding of soluble TCR was initiated by injecting the dissolved α and β chain inclusion bodies simultaneously into a vigorously stirring refolding buffer (5 M urea, 0.4 M l-arginine, 100 mM Tris pH 8.1, 6.5 mM cysteamine-HCl, 3.7 mM cystamine di-hydrochloride) chilled to 4°C, to a final concentration of 60 mg/L.

The solution was left for 3 h, then dialyzed for 24 h against ten volumes of demineralized water, followed by ten volumes of 10 mM Tris pH 8.1. All dialysis steps were carried out at 4°C. Dialyzed TCR was isolated from impurities by filtering and loading onto a POROS 50 HQ anion exchange column (Applied Biosystems). The column was washed with 10 mM Tris pH 8.1 and bound protein was eluted with a NaCl gradient (0–1 M) in the same buffer. Correctly refolded protein was confirmed by reduced and non-reduced sodium dodecyl sulfate (SDS)-PAGE. Fractions containing correctly refolded TCR were pooled, concentrated and further purified on a Superdex 200 gel filtration column (Amersham Biosciences, Uppsala, Sweden) in HEPES-buffered saline (HEPES pH 7.4, 150 mM NaCl, 3 mM EDTA). The final purified c23 TCR was analyzed by SDS-PAGE in reducing and non-reducing conditions. Peak fractions were pooled and concentrated prior to BIAcore™ surface plasmon resonance studies.

### Measurement of CTL degranulation, cytokine and chemokine release

Antigen-presenting cells (2×10^4^ Hmy.2 C1R B cells transfected with HLA-A68) were pre-pulsed with the indicated concentrations of peptide and washed twice with serum-free RPMI medium. CTL (5×10^3^) were added in each assay well and incubated for 4 h at 37°C in 96-well plates in presence of fluorescein isothiocyanate-conjugated anti-CD107a mAb (BD Pharmingen) and Golgi-stop (BD Pharmingen) diluted 1 in 100 and 1 in 3000, respectively. Cells were pelleted by centrifugation and separated from the supernatants before staining with anti-CD8 mAb directly conjugated to allophycocyanin (Becton Dickinson) and 7-amino-actinomycin dye (Becton Dickinson). Supernatants were assayed by human Th1/Th2 cytokine cytometric bead array for IL-2, IL-4, IL-6, IL-10, IFN-γ and TNF-α secretion, and chemokine cytometric bead array for monocyte chemotactic protein-1, MIP-1α, MIP-1β, IL-8 and RANTES secretion (BD Pharmingen) according to the manufacturer's instructions. Analysis was performed using a FACSCalibur (Becton Dickinson) flow cytometer. IFN-γ ELISPOT assays and chemokine ELISA were conducted as described previously 13, 20.

## Abbreviations:

MHCI: MHC class I
pMHCI: peptide-MHC class I
